# A pan-cancer analysis of the prognostic and immunological roles of zinc finger protein 514 in human tumors

**DOI:** 10.3389/fonc.2025.1592989

**Published:** 2025-06-17

**Authors:** Shuai Zhao, Lin Cheng, Xingzhao Ji, Shengnan Sun, Yi Liu, Qiang Wan

**Affiliations:** ^1^ Shandong Provincial Key Medical and Health Laboratory of Cell Metabolism, Jinan Central Hospital, Cheeloo College of Medicine, Shandong University, Jinan, China; ^2^ Shandong Provincial Key Medical and Health Laboratory of Cell Metabolism, Central Hospital Affiliated to Shandong First Medical University, Jinan, Shandong, China; ^3^ Research Center of Translational Medicine, Central Hospital Affiliated to Shandong First Medical University, Jinan, China; ^4^ School of Medicine, Chongqing University, Chongqing, China; ^5^ Department of Pulmonary and Critical Care Medicine, Shandong Provincial Hospital Affiliated to Shandong First Medical University, Jinan, Shandong, China; ^6^ Shandong Key Laboratory of Infections Respiratory Disease, Medical Science and Technology Innovation Center, Shandong First Medical University & Shandong Academy of Medical Sciences, Jinan, Shandong, China

**Keywords:** ZNF514, pan-cancer analysis, bioinformatics, prognosis, immune infiltration

## Abstract

**Background:**

Recent studies have highlighted the essential role of the zinc finger gene family, whose encoded proteins play a pivotal role in all stages of tumor initiation and development. Zinc Finger Protein 514 (ZNF514) is a member of the ZNF family, and its abnormal expression and prognostic value in human pan-cancer have not yet been described. The purpose of this study is to investigate the prognostic and immunological roles of ZNF514 in pan-cancer and to confirm its cancer-promoting effect in renal clear cell carcinoma.

**Methods:**

In our study, we utilized the Human Protein Atlas (HPA) database to determine the expression of ZNF514 in human normal and tumor tissues. We also used the Tumor Immune Estimation Resource 2.0 (TIMER 2.0) database to investigate the association between ZNF514 expression and immune checkpoint genes and immune infiltration. To detect the expression and prognostic value of ZNF514 in pan-cancers, we utilized The Cancer Genome Atlas (TCGA) or the Genotype-Tissue Expression (GTEx) databases and analyzed the data using the Kaplan-Meier plotter, GEPIA2, cBioPortal, or Xiantao software. Additionally, we obtained the protein-protein interaction network of ZNF514 from the STRING database. To validate our findings, we performed immunohistochemistry on clinical samples. Furthermore, we conducted cellular functional experiments to examine the effects of ZNF514 overexpression or knockdown on renal clear cell carcinoma cell proliferation, migration, and invasion.

**Results:**

Our study found that ZNF514 expression was elevated in tumor tissues than in normal tissues in most tumor types. Furthermore, high expression of ZNF514 was associated with poor overall survival (OS) and disease-free survival (DFS) in certain tumor types. Further analysis of ZNF514 gene mutation data revealed that ZNF514 protein is infrequently mutated in human cancers. Moreover, ZNF514 affected prognosis and was associated with the expression of multiple immune checkpoint genes and the abundance of tumor-infiltrating immune cells across multiple types of cancer. Finally, our molecular biology experiments confirmed the oncogenic effect of ZNF514 in renal clear cell carcinoma.

**Conclusion:**

Our study revealed that ZNF514 may serve as an immunological and prognostic biomarker in multiple human cancers, especially in KIRC, LIHC, LUSC, and COAD.

## 1 Introduction

Up to now, cancer remains one of the most pressing public health issues worldwide due to its high mortality rate and significant economic burden (1, 2). While conventional therapies such as surgery, radiotherapy, chemotherapy, and targeted therapy have advanced significantly, the prognosis for most cancers remains poor (3, 4). Therefore, novel therapeutic targets are urgently required. It is meaningful to explore the clinical prognostic relevance and molecular mechanisms of genes that may be involved in tumorigenesis and progression, not to mention their pan-cancer expression.

Zinc finger protein 514 (ZNF514) belongs to Zinc finger gene family, which is the largest transcriptional regulator family in mammals, and is highly conserved evolutionarily. Multiple lines of evidence have shown that zinc finger proteins play crucial roles in development, metabolism, proliferation and cancer progression (5–9). For instance, ZNF830 promotes homologous-recombination repair and mediates cancer chemoresistance (10); ZNF281 acts as a potential diagnostic marker for oral squamous cell carcinoma (11); and ZNF419 might serve as a potential prognostic and immunological pan-cancer biomarker (12).

The aforementioned evidence highlights the potential utility of ZNF514 in cancer research and treatment. However, previous research on ZNF514 has been limited, and there has been no pan-cancer investigation into the relationship between ZNF514 expression and multiple cancers. Therefore, we utilized various bioinformatics approaches to conduct a comprehensive pan-cancer analysis of ZNF514. We obtained oncological data for ZNF514 from The Cancer Genome Atlas (TCGA) to determine the prognostic landscape of ZNF514. Additionally, we investigated the possible links between ZNF514 expression and the tumor immune microenvironment and mutation status across various cancers. Furthermore, we conducted immunohistochemical (IHC) analyses to further confirm the role of ZNF514. Our study indicates that ZNF514 is not only a marker for tumor immune microenvironment changes and poor prognosis but also a promising candidate therapeutic target for cancer treatment.

## 2 Results

### 2.1 Differential expression of ZNF514 in various human normal tissues

**Figure 1:**
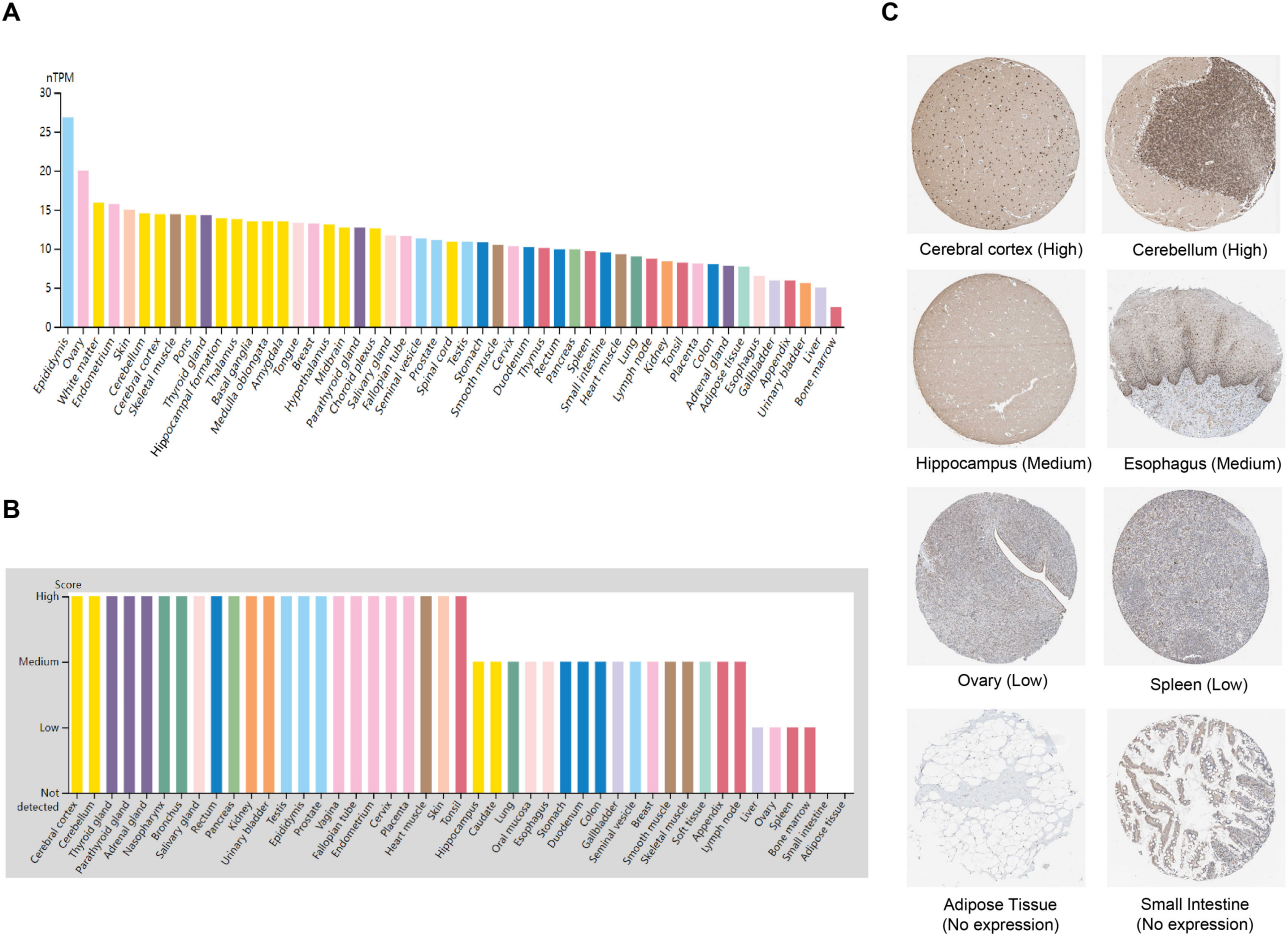
ZNF514 expression in various human normal tissues. **(A)** The mRNA expression profile of ZNF514 in normal human tissues from the HPA database. **(B)** The protein expression profile of ZNF514 in normal human tissues from the HPA database. **(C)** Representative IHC images of ZNF514 expression in the normal cerebral cortex, cerebellum, hippocampus, esophagus, ovary, spleen, adipose tissue and small intestine from the HPA database.

We investigated the mRNA and protein expression levels of ZNF514 in various normal tissue types using the human Protein Atlas (HPA) database. As depicted in **Figure 1A**, ZNF514 mRNA expression was relatively high in the epididymis, ovary, white matter, endometrium, and skin, but relatively low in the esophagus, gallbladder, appendix, urinary bladder, liver, and bone marrow. Subsequently, we examined its protein expression levels. As depicted in **Figure 1B**, ZNF514 protein expression was also higher in endometrium and skin, but was not detectable in adipose tissue or small intestine. ZNF514 mRNA and protein exhibited different expression patterns in normal tissues, such as ovary and bladder. This phenomenon may be attributed to the complexity of gene translation, which has not been experimentally validated. Immunohistochemical staining revealed that ZNF514 was predominantly localized in the nucleus, and representative tissue staining results for different expression levels were displayed in **Figure 1C**. These results included cerebral cortex (high), cerebellum (high), hippocampus (middle), esophagus (middle), ovary (low), spleen (low), adipose tissue (no expression), and small intestine (no expression).

### 2.2 ZNF514 expression in cancers

**Figure 2:**
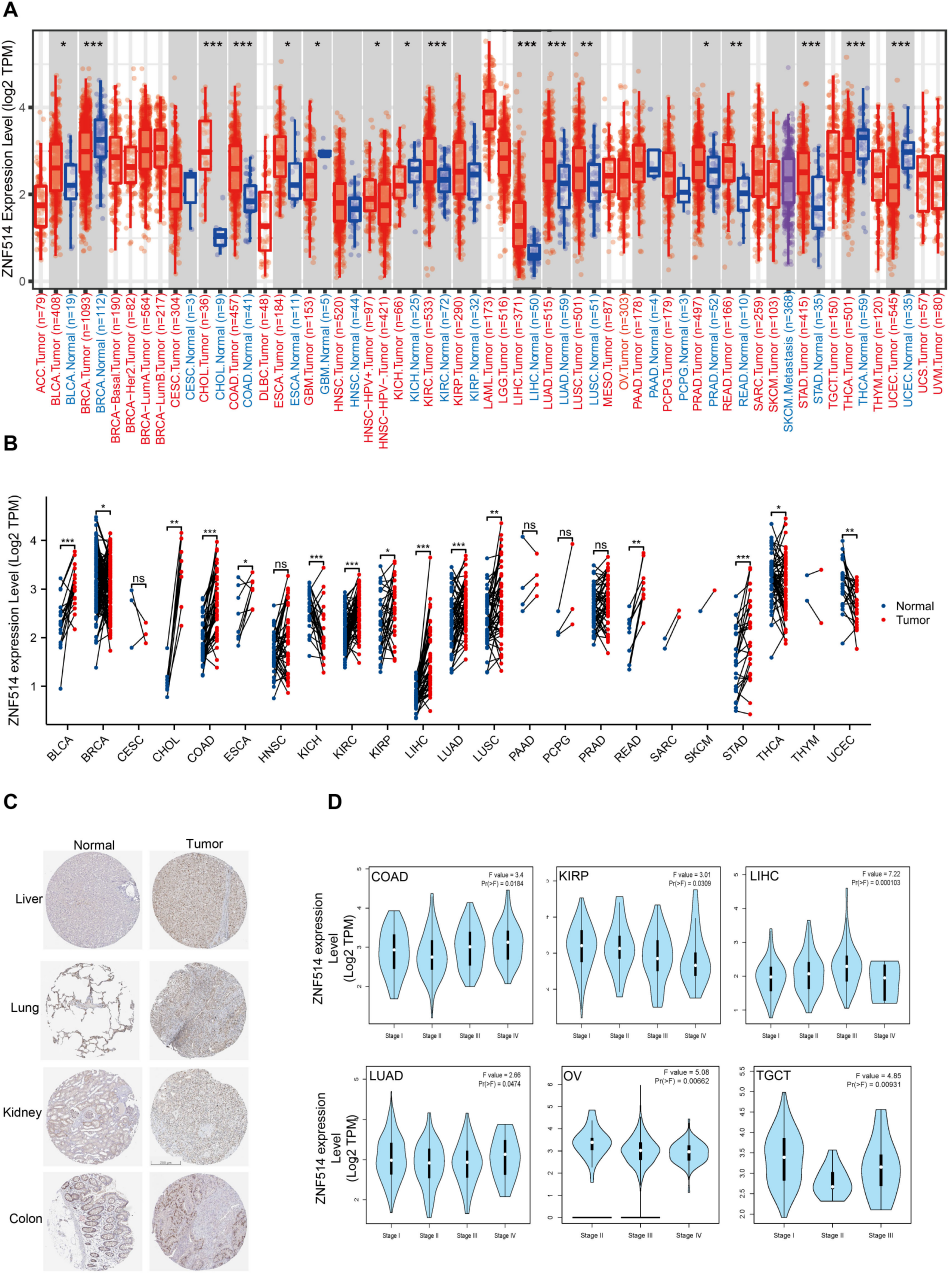
ZNF514 expression in various cancers. **(A)** The expression levels of ZNF514 in tumors and normal tissues of different cancer types in the TCGA database were analyzed using TIMER2.0 software (*P<0.05, **P<0.01, ***P<0.001). **(B)** The expression differences of ZNF514 from the TCGA databases in different tumors and normal tissues. (*P<0.05, **P<0.01, ***P<0.001). **(C)** Representative IHC images of ZNF514 expression in normal and tumor tissues from the HPA. **(D)** Association between ZNF514 expression level and patient overall survival(OS)/disease-free survival(DFS) in TCGA tumors.

To investigate the expression changes of ZNF514 in cancers, we employed TIMER 2.0 and TCGA website to evaluate the mRNA expression level of ZNF514 in tumor tissues and adjacent normal tissues. As shown in **Figure 2A**, the mRNA expression of ZNF514 was significantly increased in bladder urothelial carcinoma (BLCA), cholangiocarcinoma (CHOL), colon adenocarcinoma (COAD), esophageal carcinoma (ESCA), kidney renal clear cell carcinoma (KIRC), liver hepatocellular carcinoma(LIHC), lung adenocarcinoma (LUAD), lung squamous cell carcinoma (LUSC), prostate adenocarcinoma (PRAD), rectum adenocarcinoma (READ) and stomach adenocarcinoma (STAD) but reduced in breast invasive carcinoma (BRCA), glioblastoma multiforme (GBM), kidney chromophobe (KICH), head and neck squamous cell carcinoma(HNSC), thyroid carcinoma (THCA) and uterine corpus endometrial carcinoma (UCEC). In order to comprehensively evaluate the expression of ZNF514 in various cancer types, we will conduct a joint analysis based on the Timer2.0 database of the TCGA database and the Xiantao database, respectively analyzing the differences between tumors and normal tissues in each cancer type. The expression of ZNF514 was elevated in most cancers, including BLCA, CHOL, COAD, ESCA, GBM, KIRC, kidney renal papillary cell carcinoma (KIRP), LIHC, LUAD, LUSC, READ and STAD. In contrast, ZNF514 expression in the tumor tissues of BRCA, KICH, THCA and UCEC was significantly decreased (**Figure 2B**). The immunohistochemical staining data in the HPA database showed that the ZNF514 protein was also highly expressed in most tumors (**Figure 2C**). Additionally, we compared ZNF514 expression levels among different clinicopathological stages using the GEPIA database and observed significant associations between ZNF514 mRNA expression and tumor stages in COAD, KIRP, LIHC, LUAD, OV, and TGCT (p < 0.05, Kruskal-Wallis test) (**Figure 2D**).

### 2.3 Diagnostic and prognostic roles of ZNF514 in cancer patients

**Figure 3:**
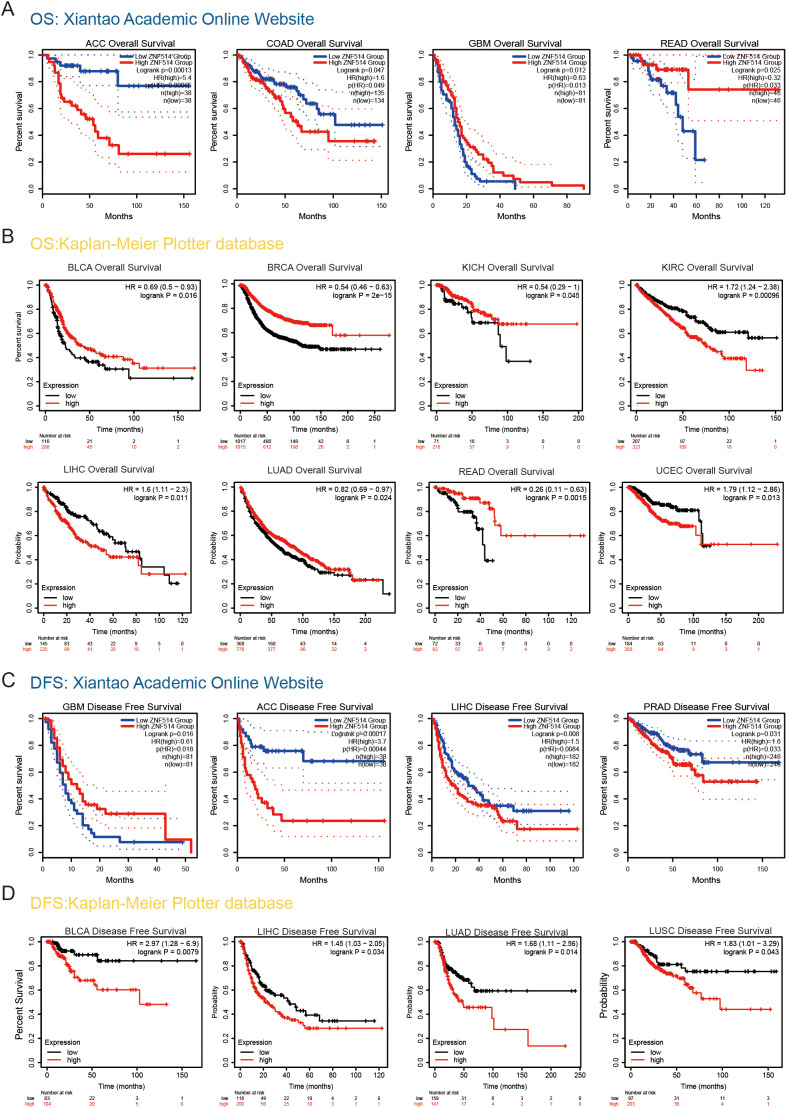
Association between ZNF514 expression level and patient overall survival (OS)/disease-free survival (DFS) in TCGA tumors. The positive results OS curves **(A, B)** were listed. The positive results of DFS curves **(C, D)** were also displayed.

To investigate the potential association between ZNF514 expression level and prognosis, we conducted a pan-cancer survival analysis with overall survival (OS) and disease free survival (DFS) using the GTEx and Kaplan–Meier survival analysis dataset. Our analysis revealed that high ZNF514 expression was associated with poor OS in 5 tumor types, such as Adrenocortical carcinoma (ACC) and KIRC. However, in other 6 tumors, high ZNF514 expression may be related to better OS (**Figures 3A, B**). Furthermore, high ZNF514 expression was related to poor DFS in ACC, BLCA, LIHC, LUSC, LUAD and PRAD. In contrast, high ZNF514 expression levels were positively associated with better prognosis in GBM (**Figures 3C, D**).To address the potential confounding effects of clinical variables, we further conducted a multivariate Cox regression analysis using the UALCAN database, including age, gender, and tumor stage. Some results indicate that ZNF514 remains an independent prognostic factor for overall survival. Although the significance of the results in other parts is not high, the overall trend is consistent with our conclusion. Even after adjusting for these covariates, this association still existed, which strengthened the clinical relevance of tumor progression.

**Figure 4:**
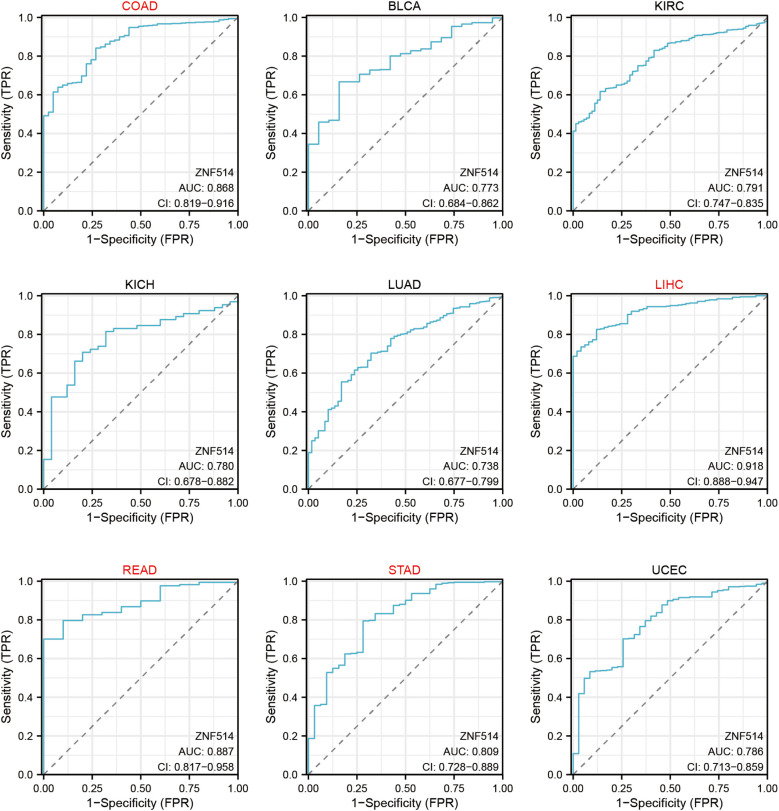
Receiver Operating Characteristics (ROC) analysis of ZNF514 gene in TCGA database.

We also utilized ROC curves to evaluate the diagnostic accuracy of ZNF514 in human tumors. The data suggested that ZNF514 had a high accuracy (AUC > 0.8) in predicting the diagnosis in COAD, LIHC, READ and STAD (**Figure 4**).

### 2.4 The genetic alteration of ZNF514 in TCGA tumor types

**Figure 5:**
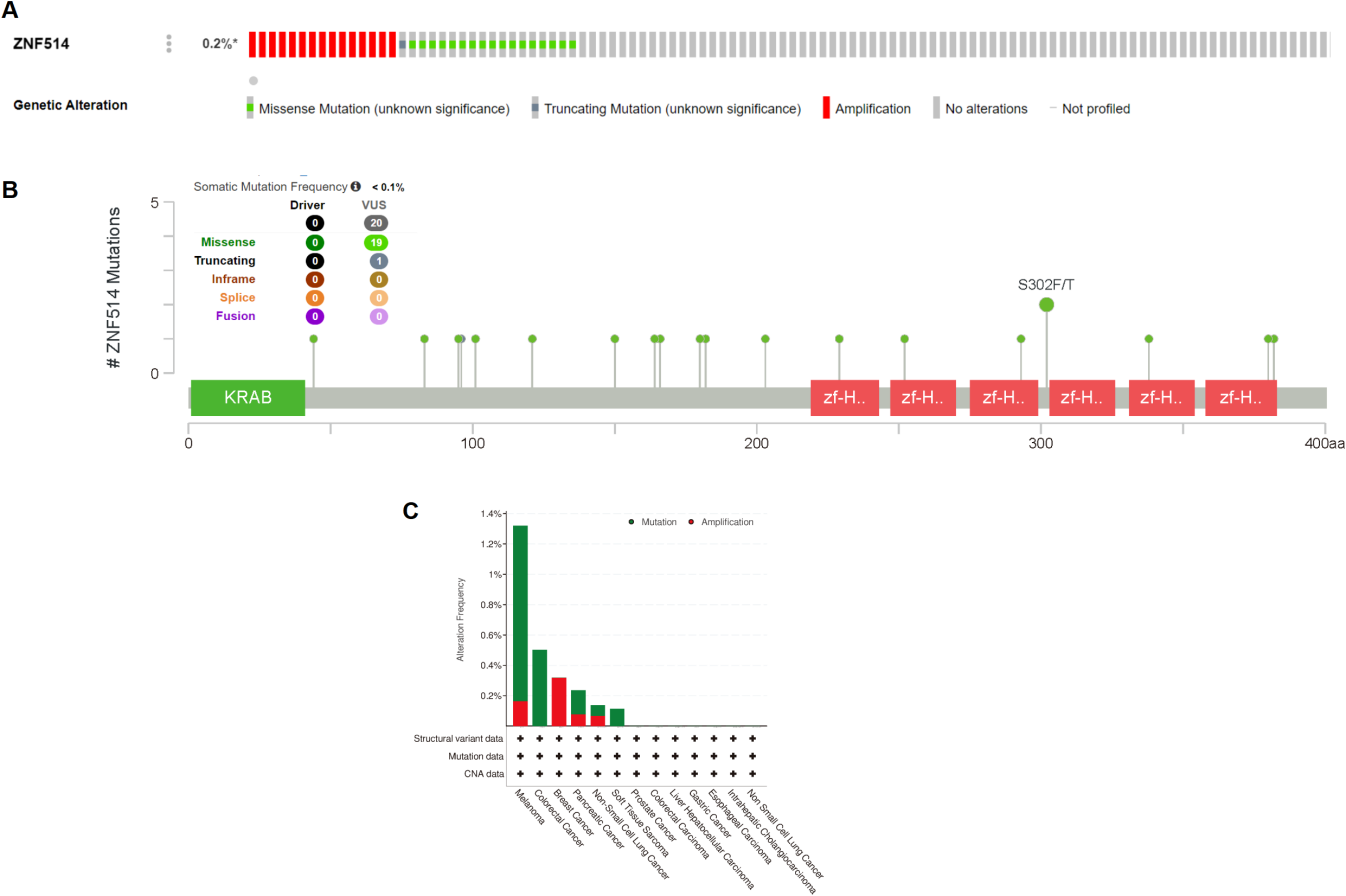
Mutation status of ZNF514 in various tumors. Mutation status of ZNF514 in TCGA tumors was analyzed using the cBioPortal tool. The alteration frequency with mutation type **(A, C)** and mutation site **(B)** are displayed.

As genetic alterations are known to be one of the key factors promoting the occurrence and development of tumors, we utilized the cBioPortal website to analyze the mutation of the ZNF514 gene in all tumor tissue data from TCGA. Our analysis revealed that ZNF514 gene alterations frequency was less than 0.2% in 75661 patients (**Figure 5A**). The most frequently mutated region (S302F/T) in the ZNF514 protein was mutated two times in total (**Figure 5B**). Missense mutation of ZNF514 accounted for the largest proportion of all mutation types, with melanoma, colorectal cancer, and pancreatic cancer having the highest occurrence rates of 1.16%, 0.5%, and 0.16%, respectively (**Figure 5C**). These findings suggest that the role of ZNF514 in the occurrence and progression of certain cancers may not be caused by gene mutations. Further research is needed to verify these findings and determine the potential mechanisms of ZNF514 in the occurrence and progression of cancer.

### 2.5 Enrichment analysis of genes related to ZNF514

**Figure 6:**
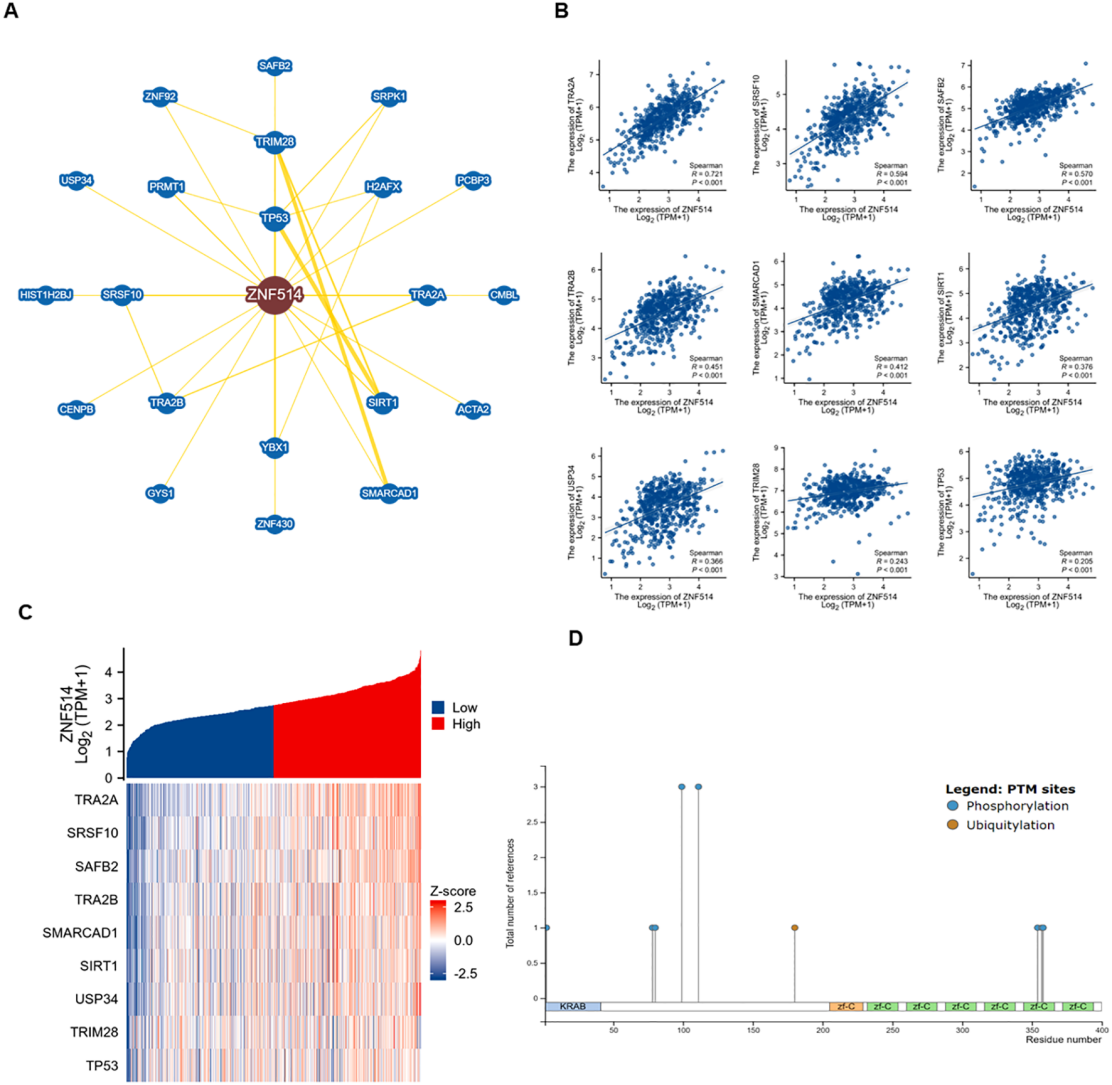
Enrichment analysis of genes related to ZNF514. **(A)** Protein-protein interaction network of ZNF514 from STRING database. **(B)** Analysis of ZNF514 Protein Interaction Network and Correlation of Key Pathways (Based on STRING Database) **(C)** Correlation analysis of ZNF514 mRNA expression with its top nine interacting genes in KIRC (TCGA dataset; Spearman’s correlation). **(D)** Predicted post-translational modification sites of ZNF514 protein, including phosphorylation and ubiquitination (K180).

Moreover, we utilized the STRING tool to obtain 20 ZNF514-interacting proteins and create a protein-protein interaction (PPI) network, which is shown in **Figure 6A**. The first nine proteins that were found to be most significantly related are shown in the **Figure 6B**, and these proteins were enriched in pre-mRNA splicing, cell cycle regulation, apoptosis, differentiation, the stress response, and regulation of immune genes. Furthermore, we validated the first nine related genes in KIRC, which likewise showed high correlation. Additionally, USP34 (Ubiquitin Specific Peptidase 34) acts as a deubiquitination enzyme and metabolism of proteins. Next, the phosphosite website shows that ZNF514 has a few phosphorylation sites and only one ubiquitination site(K180) (**Figure 6D**). Therefore, we hypothesized that USP34 might function as a deubiquitinating enzyme for ZNF514 and deubiquitinated ZNF514 at K180 site. These findings suggest that ZNF514 may interact with other proteins and be involved in various cellular processes, and further studies are necessary to elucidate the mechanisms of ZNF514 and its interacting proteins in cancer development and progression.

### 2.6 Correlation between ZNF514 expression and immune cell infiltration across tumor types

**Figure 7:**
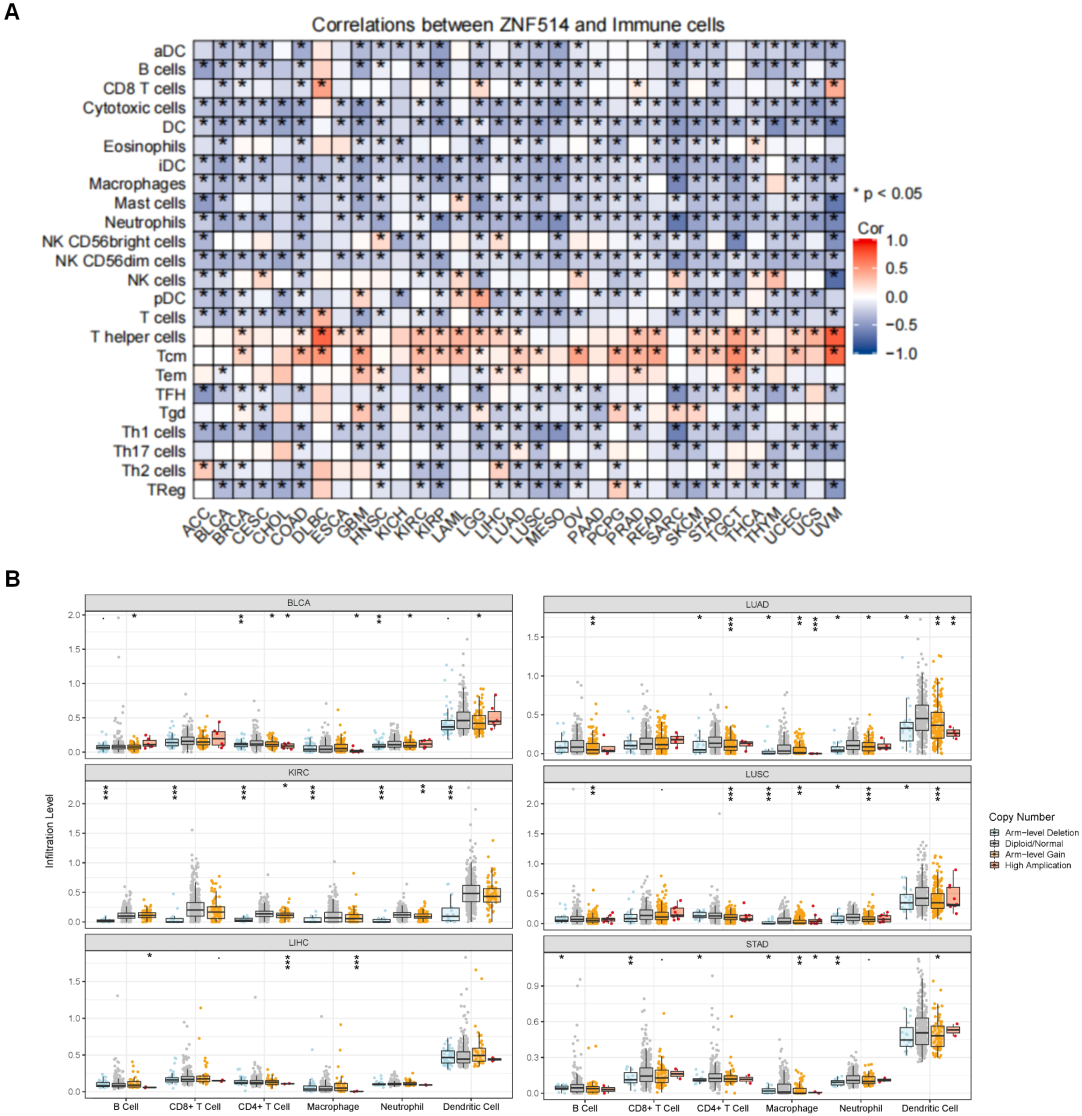
The correlation between ZNF514 expression level and immune cells. **(A)** The heat map of the correlation between ZNF514 expression and immune cell infiltration in different cancer types is from the Xiantao website. *P < 0.05. **(B)** Box plot of the correlation between ZNF514 copy number alterations (CNA) and immune cell infiltration abundance.

The response to immunotherapy in cancer patients is modulated by the tumor microenvironment (TME). The TME is primarily composed of tumor cells and tumor-infiltrating immune cells (TIICs) (13, 14).TIICs are often dysfunctional, failing to impede tumor growth and even potentially promoting its progression, ultimately leading to immune escape. The presence of immune cells is closely tied to both the initiation and progression of tumors. Consequently, we evaluated the correlation between ZNF514 expression and immune cells by utilizing the TCGA and TIMER 2.0 databases. Remarkably, the expression levels of ZNF514 exhibited a negative correlation with immune cell infiltration in a majority of tumor types, particularly with regards to dendritic cells and neutrophils (**Figures 7A, B**). These findings suggest that ZNF514 may be involved in regulating the immune response in the tumor microenvironment, and further studies are necessary to elucidate the underlying mechanisms and potential implications in cancer immunotherapy.

### 2.7 ZNF514 promoted proliferation, migration and invasion of ccRCC cells

**Figure 8:**
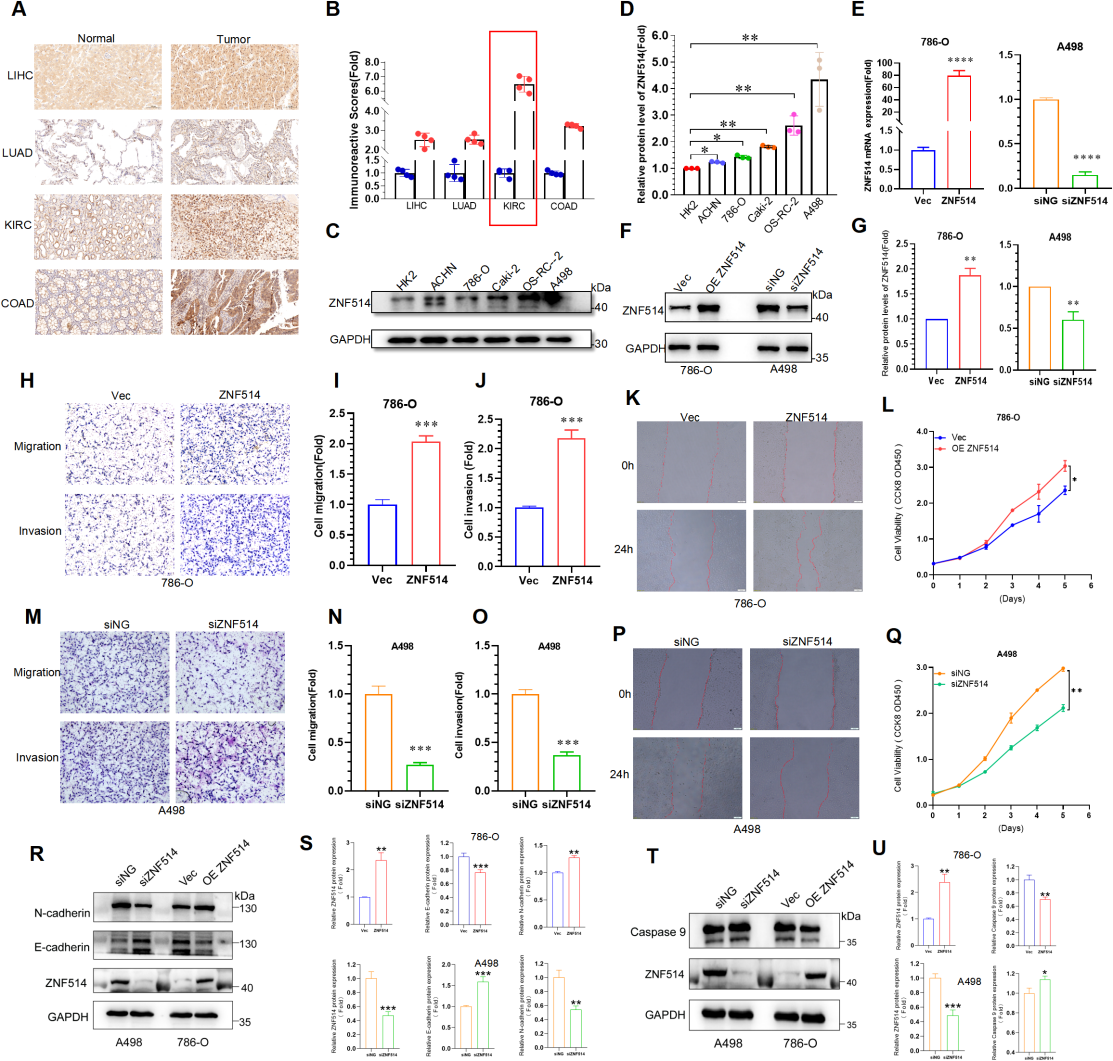
ZNF514 promoted ccRCC cell proliferation, migration and invasion. **(A)** IHC images of ZNF514 in LIHC, LUAD, KIRC, COAD and matched normal tissues. **(B)** IHC score of ZNF514 in LIHC, LUAD, KIRC, COAD and matched normal tissues. **(C, D)** The protein expression levels of ZNF514 in normal cells HK2 and ccRCC cells. **(E–G)** ZNF514 knockdown/overexpression efficiency was determined by western blotting and real-time polymerase chain reaction. **(H-J, M–O)** Transwell assays showed that ZNF514 affected ccRCC cell mobility and metastasis compared with control cells (200×). **(K, P)** Wound healing assays showed that ZNF514 involved in ccRCC cell migration compared with control cells. **(L, Q)** The proliferation ability of ccRCC cells measured by Cell Counting Kit-8. **(R, S)** The protein expression levels of E-cadherin and N-cadherin in different cell groups. **(T, U)** The protein expression levels of Caspase 9 in different cell groups.*P < 0.05, **P <0.01, ***P <0.001.

To further explore the expression of ZNF514 in pan-cancer, we collected clinical samples from 16 cancer patients in Jinan Central Hospital Affiliated to Shandong First Medical University for histochemical analysis and found that ZNF514 was overexpressed in LIHC, LUAD, KIRC and COAD, as shown in the IHC analysis (**Figure 8A**). Furthermore, it was found that the IHC score of ZNF514 in the KIRC tissue was much higher (**Figure 8B**). To evaluate the functions of ZNF514 in KIRC cell proliferation, migration, and invasion, various cellular experiments were carried out. Firstly, the expression levels of ZNF514 were determined in human permanent renal tubular epithelial cell HK2 and renal clear cell carcinoma cell lines ACHN, 786-O, Caki-2, OS-RC-2, and A498 by Western blotting (**Figure 8C**). The results showed that ZNF514 was upregulated in most ccRCC cell lines, and the expression was relatively highest in A498. While 786-O expression was higher than HK2 but lower than other ccRCC cell lines (**Figure 8D**). Therefore, Heterologous overexpression of ZNF514 was carried out in 786-O cells, and ZNF514 expression was interfered by siRNA in A498. QPCR (**Figure 8E**) and WB (**Figures 8F, G**) showed that ZNF514 expression was significantly changed in A498 and 786-O cells after transfection. The migration and invasion of 786-O cells were dramatically promoted by ZNF514 overexpression (**Figures 8H–J**). A wound-healing assay revealed that ZNF514 notably induced the wound closure rate of 786-O cells compared with Vec (**Figure 8K**). The CCK-8 assay indicated that ZNF514 markedly promoted the proliferation (**Figure 8L**). On the contrary, the migration, invasion, and proliferation of A498 cells were dramatically inhibited by ZNF514 knockdown (**Figures 8M–Q**). These data demonstrate that ZNF514 plays an important role in promoting growth and metastasis in KIRC. To further investigate the functional role of ZNF514 in renal cell carcinoma (RCC), we conducted EMT and apoptosis experiments on the 786-O (ZNF514 overexpression) and A498 (ZNF514 knockdown) cell lines. Western blot analysis showed that compared with the vector control, overexpression of ZNF514 significantly downregulated the epithelial marker E-cadherin and up-regulated the mesenchymal marker N-cadherin (**Figures 8R, S**). These results indicate that ZNF514 induces EMT, which is a key process in cancer metastasis. Meanwhile, in ZNF514-silenced A498 cells, the level of lysed Caspase-9 (active form) increased, indicating that ZNF514 mainly regulates the intrinsic (mitochondrial) apoptotic pathway (**Figures 8T, U**). Further experimental studies are necessary to validate these findings and determine the underlying molecular mechanisms of ZNF514 in KIRC cell proliferation, migration, and invasion.

## 3 Materials and methods

### 3.1 Gene expression analysis

We utilized the HPA (https://www.proteinatlas.org) database to explore ZNF514 mRNA and protein expression level in normal human tissues. The TIMER2 (tumor immune estimation resource, version 2, http://timer.cistrome.org/) database was used to analysis the expression level of ZNF514 between tumor types and adjacent normal tissues (15). The RNA-seq data were collected from the TCGA (http://cancergenome.nih.gov) and GTEx (http://commonfund.nih.gov/GTEx/) database. Furthermore, The association between ZNF514 expression and tumor stage were analyzed by the Gene Expression Profiling Interactive Analysis (GEPIA; http://gepia.cancer-pku.cn/) database.

### 3.2 Survival analysis

We verified the prognostic value of ZNF514 based on clinical data from the TCGA and GTEx database, Xiantao Academic Online Website (https://www.xiantao.love/) and Kaplan-Meier Plotter database(http://kmplot.com/) were used for bioinformatics analysis based on the R language. The statistical significance of OS and DFS between the high and low ZNF514 expression groups in patients with 26 cancer types were analyzed. We used a 50% cut-off value to segment the high and low expression groups, and the Log-rank test was used for hypothesis testing. Statistical significance was set at P < 0.05.

### 3.3 ROC curve analysis

We obtained RNA-seq data from TCGA and GTEx database, Xiantao Academic Online Website (https://www.xiantao.love/) were used for bioinformatics analysis based on the R language.

### 3.4 Genetic alteration analysis

The cBioPortal tool was employed to analyze ZNF514 genetic alterations. Alteration frequency and type and mutated site for ZNF514 from TCGA Pan-Cancer Atlas Studies were obtained and analyzed. We input ‘ZNF514’ to ‘Quick Search’ module, and genetic alterations and mutated site data can be obtained from ‘Cancer Types Summary’ and ‘Mutations’ modules.

### 3.5 Immune infiltration analysis

The association data between ZNF514 expression and immune cells were obtained from the “GENE” module in the TIMER (https://cistrome.shinyapps.io/timer/) database.

### 3.6 Protein network and ZNF514-related gene enrichment analysis

STRING website was utilized to find a human ZNF514-binding protein co-expression network. Next, we set the main parameters: max number of interactors to show (‘no more than 20 interactors’).

### 3.7 Patients and tissue specimens

All clinical samples were obtained from the Jinan Central Hospital Affiliated to Shandong First Medical University, China. All samples were collected with the consent of the patients and the study was approved by the Ethics Committee of the Jinan Central Hospital. All patient specimens and clinical data used in this study complied with the principles of the Declaration of Helsinki.

### 3.8 Immunohistochemistry staining

To evaluate differences in ZNF514 expression at the protein level, IHC images of ZNF514 protein expression in normal tissues and tumors tissues were downloaded from the HPA (Human Protein Atlas) (http://www.proteinatlas.org/). Furthermore, we collected the adjacent to cancer and tumor tissue from LIHC, LUAD, KIRC and COAD patients. A total of 16 patients were included. Among them, 4 cases each from LIHC, LUAD, KIRC and COAD patients were selected for analysis. For IHC analysis, these paraffin-embedded slides were deparaffinized and rehydrated using xylene and a graded series of ethanol (100%, 95%, 80%, 75%), and then washed with PBS three times for 5 min each time. Subsequently, citrate antigen restore solution was used to repair antigens on slices in a microwave oven at the heat preservation for 18 min, followed by natural cooling, and washed with PBS three times for 5 min each time. The slides were then immersed in 3% H_2_O_2_ solution at room temperature to eliminate endogenous peroxidase activity. The slides were incubated in 5% BSA to block non-specific binding of antibody for 1 h and then incubated in a humidified chamber overnight at 4°C with the primary antibodies anti-ZNF514 (1:100 dilution; Immunoway; YT6669; China). This was followed by washing with PBS and incubation with a secondary antibody for 60 min at 37°C. The slices were washed with PBS after incubation. For a color reaction, slides were incubated with the DAB solution. Subsequently, the slides were then counterstained with hematoxylin, dehydrated with graded alcohol series, and covered-slipped with neutral balsam. The slides were imaged using a microscope slide scanner and analyzed by Qupath (https://github.com/qupath/qupath/releases).

### 3.9 Cell lines and cell culture

The HK2, ACHN, 786-O, Caki-2, OS-RC-2 and A498 cells were purchased from Procell (Procell Life Science & Technology Co., Ltd., Wuhan, China). By STR identification, the cell line was authenticated. Cells were cultured in PRMI-1640 medium (Gibco, Thermo Fisher Scientific), supplemented with 10% fetal bovine serum (Sijiqing, Hangzhou, China), and then maintained at 37°C with 5% CO_2_.

### 3.10 Transfection assay

The pcDNA3.1 vector was used to construct the ZNF514 overexpression plasmid and control the overexpression plasmid. The plasmids were constructed by WZ Biosciences Inc. (Shandong, China). Subsequently, exponentially growing untreated cells were cultured for 24 h before transfection. pcDNA3.1-ZNF514 was transiently transfected with 786-O cells using Lipofectamine 3000 (Invitrogen, USA).

SiRNAs targeting ZNF514 (F: 5′-CAGGUAGUAUCAGUGGAAATT-3′; R: 5′-UUUCCACUGAUACUACCUGTT-3′) were synthesized by RiboBio(Guangzhou, China). A498 cells in 6-well plates were transfected with siRNA using riboFECTTM CP Transfection kit (RiboBio, Guangzhou, China) according to the manufacturer’s instructions.

### 3.11 Western blotting

Total protein was extracted from the 786-O and A498 cells using the RIPA buffer (Beyotime, China). Total proteins were separated in SDS-PAGE and then transferred onto PVDF membranes. After blocking with 5% non-fat milk for 1.5 h at room temperature, the membranes were incubated overnight with the primary antibodies ZNF514 (1:100 dilution; Immunoway; YT6669; China) at 4°C. Subsequently, the membranes were incubated with the corresponding secondary antibodies and observed under enhanced chemiluminescence (ECL, Thermo Fisher Scientific).

### 3.12 Real-time polymerase chain reaction

Total RNA was extracted from treated cells using TRIzol Reagent (Invitrogen, Shanghai, China) according to the manufacturer’s instructions. 1μg mRNA was reversed-transcribed to cDNA with PrimeScript™ RT reagent Kit (Perfect Real Time) (TAKARA, Beijing, China). Quantitative realtime PCRs were performed in triplicates using ABGENE PCR STRIPS (Thermo, Shanghai, China).Each PCR reaction mixture contained 1μl cDNA, 3.6μl RNase-free water, 5μl TB Green II(TAKARA, Beijing, China) and 0.2μl of each sense and anti-sense primers per well. Thermal cycling conditions were 30s at 95˚C, then 40 cycles of 95˚C for 5s and 60˚C for 30s, followed by 95˚C for 5s and 60˚C for 1 min on QuantStudioTM Real-Time PCR Instrument (Thermo Fisher Scientific, Shanghai, China). Each targeting gene expression was normalized with β-actin. The primers (ZNF514 F: 5’-ACAAATCTGCCACCACCCTTA-3’; R: 5’-TGTTTCCCCCTAAAGTCTGCC-3’) were obtained from BIOSUNE (Shanghai, China).

### 3.13 CCK-8 cell viability assays

Cells were seeded into 96-well plates (3000 cells/well) with 100 μL medium and incubated overnight and then cultured for 1, 2, 3, 4, 5 or 6 days. 10 μL CCK-8 reagent (MedChem Express, Monmouth Junction, NJ, USA) was added to each well. After 2 h of incubation, the OD values were determined using a microplate reader (SpectraMax i3x, USA) at a wavelength of 450 nm. Experiments were performed in triplicates.

### 3.14 Transwell assays

Transwell assays were performed to assess cell invasion and migration abilities using 24-well plates with BD chambers (8-mm pores; BD Biosciences, Shanghai, China). About 1x10^4^ (migration),4 x10^4^ (invasion) cells/well were seeded in the upper chamber and cultured in serum-free medium. Medium with 10% serum was placed in the lower chambers. After migration through the transwell membrane, the cells were fixed with 4% paraformaldehyde and stained with crystal violet (Solarbio, Beijing, China). The difference between the migration and invasion assays was the transwell chambers for migration assays were not coated with Matrigel. All transwell treatments were conducted in triplicates.

### 3.15 Wound-healing assay

Transfected cells were seeded into 6-well plates, and then a scratch wound was made using the pipette tip. The dish was washed with PBS to remove detached cells, and cells were cultured in a low-concentration serum medium. Photographs were collected at 0 and 24 h.

### 3.16 Statistical analysis

Continuous data following a normal distribution was analyzed by Student’s t test or Welch’s t test; continuous data with a non-normal distribution was assessed by Mann-Whitney U test. Chi-squared test was used for dichotomous variable and categorical data. Data are presented in bar plots as mean± standard deviation (SD) of at least three independent experiments. A p-value of <0.05 (two-tailed) was considered statistically significant. All statistical analyses were performed using Prism 8.0(GraphPad Software, San Diego, CA).

## 4 Discussion

Cancer remains a challenging and life-threatening disease, often associated with high mortality rates, poor prognosis, and significant financial burden on public healthcare systems (1). Tumor metastasis is the most significant challenge in the clinical treatment of cancer, as most cancer-related deaths are associated with disseminated disease rather than the primary tumor (16–18). However, the mechanisms underlying tumor metastasis remain unclear.ZNF514 has not been widely studied. However, various studies reported that ZNFs could function as diagnostic and prognostic biomarkers in several cancers. For instance, ZNF830 has been identified as a HR repair regulator in DNA end resection, leading to chemoresistance to genotoxic therapy (10). AKT signaling has been shown to promote ZNF322A protein stability and transcriptional activity, which in turn positively regulates transcription of alpha-adducin (ADD1) and cyclin D1 (CCND1) to promote tumorigenicity in lung cancer (19). Overexpression of zinc finger protein 384 (ZNF384), a poor prognostic predictor, has been found to promote cell growth by upregulating the expression of Cyclin D1 in hepatocellular carcinoma (20). ZNF300, a novel zinc finger protein identified specifically in humans, has been shown to promote tumor development by modulating the NF-κB pathway (21). However, there is a lack of systematic studies on ZNF514 across different types of cancer.

Our findings indicated that ZNF514 was significantly overexpressed in 11 types of cancer. The results for BLCA, CHOL, COAD, ESCA, GBM, KIRC, LIHC, LUAD, LUSC, READ, and STAD were consistent with the Genotype-Tissue Expression (GTEx) and TCGA databases. However, the expression of ZNF514 in KIRP and PRAD was not consistent in the two databases, possibly due to the small sample size. Moreover, we found a significant difference in ZNF514 expression between cholangiocarcinoma cancer tissues and normal control tissues, which may be attributed to the differences in the tumor samples. Our analysis also suggested the prognostic value of ZNF514 by analyzing OS, DFS, and ROC in different cancers. However, the exact role of ZNF514 in predicting prognosis remains unclear. We observed that high expression of ZNF514 was associated with a good prognosis in some cancers, while in others, low expression of ZNF514 was correlated with a poor prognosis. For instance, high ZNF514 expression levels were related to poor OS and DFS in ACC patients, whereas ZNF514 appeared to be a protective factor in READ. In LUAD, low ZNF514 expression was related to good DFS but poor OS. While our initial univariate analysis provided evidence for the association between [ZNF514] and tumor proliferation, the subsequent multivariate analysis accounting for age, gender, and tumor stage strengthens the validity of our findings. Nevertheless, future studies with larger cohorts and additional covariates (e.g., treatment history) are warranted.

Moreover, we analyzed the association of ZNF514 expression with TNM staging status, as the TNM staging system is still a powerful tool for evaluating and predicting the prognosis of multiple cancers (22). Our results showed a significant relationship between ZNF514 and TNM staging in COAD and KIRP, but the prognostic outcome was opposite for these two types of cancer. This may shed light on the dominant malignant phenotype of ZNF514 in tumor development or metastasis. This suggests that the role of ZNF514 may not be the same in different types of cancer, and the therapeutic emphasis may vary at different cancer stages. In addition, these findings also clearly demonstrate that ZNF514 can be utilized as a biomarker to determine the prognosis of various cancers. However, the TNM staging system also has limitations in elucidating genetic variations, and the powerful heterogeneity present among patients with the same clinical stage may lead to different clinical outcomes.

Hence, we conducted the following analyses on genetic and epigenetic regulation as well as immunity to more fully elucidate the role of ZNF514. Tumor heterogeneity, mediated by genetic (or epigenetic) alterations or caused by evolutionary selection of tumor clones and subclones, leads to molecular biological or genetic changes in tumor progeny cells, ultimately resulting in differences in tumor growth rate, invasiveness, drug sensitivity, prognosis, and other aspects (23, 24). Our study showed that ZNF514 expression was associated with immune cells in most cancer species. This may suggest that the level of ZNF514 expression affects tumor heterogeneity at genetic or epigenetic level and changes the immune microenvironment of cancers.

To date, only three report has been published about ZNF514, which suggests that ZNF514 may play a carcinogenic role in non-small cell lung cancer (25). Little is known about the function and molecular mechanisms of this gene. Gene Ontology (GO) annotations related to ZNF514 include nucleic acid binding and DNA-binding transcription factor activity (https://www.genecards.org/). Our findings indicate that USP34 protein (Ubiquitin Specific Peptidase 34) may interacts with ZNF514. USP34 works as deubiquitination enzyme and plays a role in protein metabolism. Previous studies have reported that USP34 is closely associated with the development and progression of human cancers (26–28). For example, USP34 overexpression promoted PANC-1 cell proliferation and migration via up-regulating the proteins of p-AKT and p-PKC (28). Knockdown of USP34 could significantly inhibit laryngeal squamous cell carcinoma (LSCC) cell growth, but overexpression of SOX2 could reverse this effect. Indicating that targeting USP34/SOX2 axis may be a promising strategy for the treatment of LSCC (26). Furthermore, Inhibition of USP34 induces epithelial-mesenchymal transition and promotes stemness in mammary epithelial cells (27). According to the phosphosite mimic website, ZNF514 has a few phosphorylation sites and only one ubiquitination site(K180) (**Figure 6D**). Therefore, we hypothesized that USP34 might function as a deubiquitinating enzyme for ZNF514 and deubiquitinate ZNF514 at K180 site. This hypothesis needs to be validated by further biological experiments to confirm whether USP34 is a potential upstream target for ZNF514.

IHC analysis revealed that ZNF514 was overexpressed in LIHC, LUAD,KIRC and COAD (**Figure 8A**). Additionally, the IHC score of ZNF514 was much higher in KIRC tissues (**Figure 8B**). Renal cell carcinoma (RCC) is one of the most prevalent cancers worldwide, with metastasis being the leading cause of mortality. According to cancer statistics in the United States, there are approximately 65,000 new diagnoses and 15,000 deaths each year (1). Clear cell renal cell carcinoma (ccRCC), which accounts for over 80% of sporadic RCC histopathological types, has a worse survival outcome compared to other subtypes (29). Currently, ccRCC can be treated with surgery, targeted therapy, and immunotherapy. However, clinical outcomes remain limited, and metastatic renal cancer is difficult to cure (30). Therefore, identifying key molecules involved in renal cell carcinoma is essential and highly demanded to improve clinical outcomes. Therefore, the biological function of ZNF514 in ccRCC was verified using various cellular experiments. ZNF514 knockdown markedly inhibited ccRCC cell proliferation, migration, and invasion. These results provide insight into the function of ZNF514 in ccRCC progression.

There are also limitations to this study. Firstly, some of our results are based on a single method or database and lack validation from multiple sources. Additionally, our experimental results demonstrate the relationship between ZNF514 and ccRCC, but the molecular mechanisms and its impact on the immune process are still unclear. Overall, our study has elucidated the association between ZNF514 expression and clinical prognosis as well as immune infiltration. We also utilized biological experiments to validate the cancer-promoting effect of ZNF514 in ccRCC. This study provides a theoretical basis for introducing ZNF514 as a new prognostic and immunological biomarker.

## Data Availability

Publicly available datasets were analyzed in this study. This data can be found here: HPA (https://www.proteinatlas.org), TIMER2 (tumor immune estimation resource, version 2, http://timer.cistrome.org/), TCGA (http://cancergenome.nih.gov), GTEx (http://commonfund.nih.gov/GTEx/) and GEPIA (http://gepia.cancer-pku.cn/).

## References

[1] Siegel RL Miller KD Wagle NS Jemal A. Cancer statistics, 2023. CA Cancer J Clin. (2023) 73:17–48. doi: 10.3322/caac.21763 36633525

[2] Xia C Dong X Li H Cao M Sun D He S. Cancer statistics in China and United States, 2022: profiles, trends, and determinants. Chin Med J (Engl). (2022) 135:584–90. doi: 10.1097/CM9.0000000000002108 PMC8920425 35143424

[3] Barry ST Gabrilovich DI Sansom OJ Campbell AD Morton JP. Therapeutic targeting of tumour myeloid cells. Nat Rev Cancer. (2023) 23(4):216–37. doi: 10.1038/s41568-022-00546-2 36747021

[4] Wu L Sun S Qu F Liu X Sun M Pan Y. ASCL2 affects the efficacy of immunotherapy in colon adenocarcinoma based on single-cell RNA sequencing analysis. Front Immunol. (2022) 13:829640. doi: 10.3389/fimmu.2022.829640 35774798 PMC9237783

[5] Vinjamur DS Yao Q Cole MA McGuckin C Ren C Zeng J. ZNF410 represses fetal globin by singular control of CHD4. Nat Genet. (2021) 53:719–28. doi: 10.1038/s41588-021-00843-w PMC8180380 33859416

[6] Bosanac I Wertz IE Pan B Yu C Kusam S Lam C. Ubiquitin binding to A20 ZnF4 is required for modulation of NF-κB signaling. Mol Cell. (2010) 40:548–57. doi: 10.1016/j.molcel.2010.10.009 21095585

[7] Zhao P Wang Z Xie X Jiang T Lai NC Yang B. Directed conformational switching of a zinc finger analogue regulates the mechanosensing and differentiation of stem cells. Angew Chem Int Ed Engl. (2022) 61:e202203847. doi: 10.1002/anie.202203847 36195782

[8] Singh JK Smith R Rother MB de Groot AJL Wiegant WW Vreeken K. Zinc finger protein ZNF384 is an adaptor of Ku to DNA during classical non-homologous end-joining. Nat Commun. (2021) 12:6560. doi: 10.1038/s41467-021-26691-0 34772923 PMC8589989

[9] Stevens SJ Van Essen AJ Van Ravenswaaij CM Elias AF Haven JA Lelieveld SH. Truncating *de novo* mutations in the Krüppel-type zinc-finger gene ZNF148 in patients with corpus callosum defects, developmental delay, short stature, and dysmorphisms. Genome Med. (2016) 8:131. doi: 10.1186/s13073-016-0386-9 27964749 PMC5155377

[10] Chen G Chen J Qiao Y Shi Y Liu W Zeng Q. ZNF830 mediates cancer chemoresistance through promoting homologous-recombination repair. Nucleic Acids Res. (2018) 46:1266–79. doi: 10.1093/nar/gkx1258 PMC5814808 29244158

[11] Starzyńska A Sobocki BK Sejda A Sakowicz-Burkiewicz M Szot O Jereczek-Fossa BA. ZNF-281 as the potential diagnostic marker of oral squamous cell carcinoma. Cancers (Basel). (2021) 13(11):2661. doi: 10.3390/cancers13112661 34071380 PMC8197962

[12] Zhu W Feng D Shi X Li D Wei Q Yang L. A pan-cancer analysis of the oncogenic role of zinc finger protein 419 in human cancer. Front Oncol. (2022) 12:1042118. doi: 10.3389/fonc.2022.1042118 36578929 PMC9791222

[13] Anderson NR Minutolo NG Gill S Klichinsky M. Macrophage-based approaches for cancer immunotherapy. Cancer Res. (2021) 81:1201–8. doi: 10.1158/0008-5472.CAN-20-2990 33203697

[14] Wu L Sun S Qu F Sun M Liu X Sun Q. CXCL9 influences the tumor immune microenvironment by stimulating JAK/STAT pathway in triple-negative breast cancer. Cancer Immunol Immunother. (2022) 72(6):1479–92. doi: 10.1007/s00262-022-03343-w PMC10992228 36472587

[15] Li T Fu J Zeng Z Cohen D Li J Chen Q. TIMER2.0 for analysis of tumor-infiltrating immune cells. Nucleic Acids Res. (2020) 48:W509–w514. doi: 10.1093/nar/gkaa407 32442275 PMC7319575

[16] Li Q Han Y Wang C Shan S Wang Y Zhang J. MicroRNA-125b promotes tumor metastasis through targeting tumor protein 53-induced nuclear protein 1 in patients with non-small-cell lung cancer. Cancer Cell Int. (2015) 15:84. doi: 10.1186/s12935-015-0233-x 26388699 PMC4573481

[17] Sun S Chen H Sun L Wang M Wu X Xiao ZXJ. Hotspot mutant p53-R273H inhibits KLF6 expression to promote cell migration and tumor metastasis. Cell Death Dis. (2020) 11:595. doi: 10.1038/s41419-020-02814-1 32733026 PMC7393383

[18] García-Pardo M Chang A Schmid S Dong M Brown MC Christiani D. Respiratory and cardiometabolic comorbidities and stages I to III NSCLC survival: A pooled analysis from the international lung cancer consortium. J Thorac Oncol. (2023) 18:313–23. doi: 10.1016/j.jtho.2022.10.020 PMC10463560 36396063

[19] Liao SY Kuo IY Chen YT Liao PC Liu YF Wu HY. AKT-mediated phosphorylation enhances protein stability and transcription activity of ZNF322A to promote lung cancer progression. Oncogene. (2019) 38:6723–36. doi: 10.1038/s41388-019-0928-x 31399647

[20] He L Fan X Li Y Chen M Cui B Chen G. Overexpression of zinc finger protein 384 (ZNF 384), a poor prognostic predictor, promotes cell growth by upregulating the expression of Cyclin D1 in Hepatocellular carcinoma. Cell Death Dis. (2019) 10:444. doi: 10.1038/s41419-019-1681-3 31168049 PMC6551341

[21] Wang T Wang XG Xu JH Wu XP Qiu HL Yi H. Overexpression of the human ZNF300 gene enhances growth and metastasis of cancer cells through activating NF-kB pathway. J Cell Mol Med. (2012) 16:1134–45. doi: 10.1111/j.1582-4934.2011.01388.x PMC4365892 21777376

[22] Hu D Liu Z Chen S Huang Y Zeng W Wei W. Assessment of the novel, practical, and prognosis-relevant TNM staging system for stage I-III cutaneous melanoma. Front Oncol. (2022) 12:738298. doi: 10.3389/fonc.2022.738298 35574383 PMC9104117

[23] Mcgranahan N Swanton. Clonal Heterogeneity C. and tumor evolution: past, present, and the future. Cell. (2017) 168:613–28. doi: 10.1016/j.cell.2017.01.018 28187284

[24] Pe’er D Ogawa S Elhanani O Keren L Oliver TG Wedge D. Tumor heterogeneity. Cancer Cell. (2021) 39:1015–7. doi: 10.1016/j.ccell.2021.07.009 34375606

[25] Maiuthed A Prakhongcheep O Chanvorachote P. Microarray-based analysis of genes, transcription factors, and epigenetic modifications in lung cancer exposed to nitric oxide. Cancer Genomics Proteomics. (2020) 17:401–15. doi: 10.21873/cgp.20199 PMC7367602 32576585

[26] Dai WL Yuan SX Cao JP. The deubiquitinase USP34 stabilizes SOX2 and induces cell survival and drug resistance in laryngeal squamous cell carcinoma. Kaohsiung J Med Sci. (2020) 36:983–9. doi: 10.1002/kjm2.v36.12 PMC11896225 32783291

[27] Oh E Kim JY Sung D Cho Y Lee N An H. Inhibition of ubiquitin-specific protease 34 (USP34) induces epithelial-mesenchymal transition and promotes stemness in mammary epithelial cells. Cell Signal. (2017) 36:230–9. doi: 10.1016/j.cellsig.2017.05.009 28499884

[28] Gu Z Lin C Hu J Xia J Wei S Gao D. USP34 regulated human pancreatic cancer cell survival via AKT and PKC pathways. Biol Pharm Bull. (2019) 42:573–9. doi: 10.1248/bpb.b18-00646 30686807

[29] Jonasch E Walker CL Rathmell. Clear cell renal cell carcinoma ontogeny WK. and mechanisms of lethality. Nat Rev Nephrol. (2021) 17:245–61. doi: 10.1038/s41581-020-00359-2 PMC8172121 33144689

[30] Wang J Huang F Zhao J Huang P Tan J Huang M. Tumor-infiltrated CD8+ T cell 10-gene signature related to clear cell renal cell carcinoma prognosis. Front Immunol. (2022) 13:930921. doi: 10.3389/fimmu.2022.930921 35812454 PMC9263606

