# Supplementary material for: A pan-cancer analysis of the prognostic and immunological roles of zinc finger protein 514 in human tumors
Source: Front Oncol. 2025 Jun 17;15:1592989. doi: 10.3389/fonc.2025.1592989 (PMC12209179; doi:10.3389/fonc.2025.1592989)
Supplement: Supplementary file 1 [file DataSheet1.pdf]

Supplementary Material

This PDF file includes:

1. **Supplementary Figures**

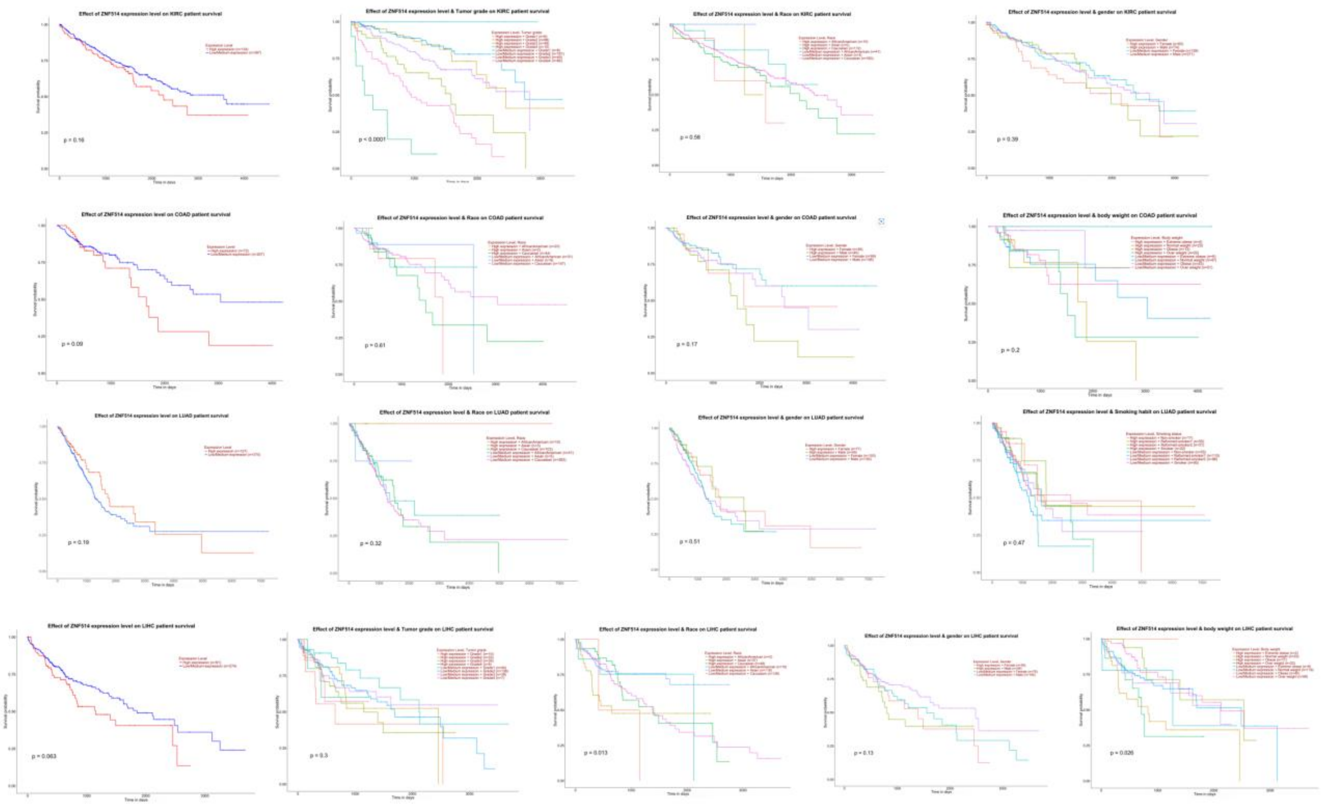

Supplementary Figure S1

Supplementary Figure S1. Multivariate Cox regression survival curves adjusted for age, gender and tumor stage from the UALCAN database.
